# An Adaptive Nonlocal Mean Filter for PolSAR Data with Shape-Adaptive Patches Matching

**DOI:** 10.3390/s18072215

**Published:** 2018-07-10

**Authors:** Peng Shen, Changcheng Wang, Han Gao, Jianjun Zhu

**Affiliations:** 1 School of Geosciences and Info-Physics, Central South University, Changsha 410083, China; shen-peng@csu.edu.cn (P.S.); dawnhan314@csu.edu.cn (H.G.); zjj@csu.edu.cn (J.Z.); 2 Key Laboratory of Metallogenic Prediction of Nonferrous Metals and Geological Environment Monitoring, Ministry of Education, Central South University, Changsha 410083, China

**Keywords:** polarimetric synthetic aperture radar (PolSAR), polarimetric likelihood ratio test (PolLRT), region growing (RG), shape-adaptive (SA) patches matching, nonlocal means (NLM)

## Abstract

The traditional nonlocal filters for polarimetric synthetic aperture radar (PolSAR) images are based on square patches matching to obtain homogeneous pixels in a large search window. However, it is still difficult for the regular patches to work well in the complex textured areas, even when the patch size has a small enough setting (e.g., 3 × 3 windows). Therefore, this paper proposes an adaptive nonlocal mean filter with shape-adaptive patches matching (ANLM) for PolSAR images. Mainly, the shape-adaptive (SA) matching patches are constructed by combining the polarimetric likelihood ratio test for coherency matrices (PolLRT-CM) and the region growing (RG), which is called PolLRT-CMRG. It is used to distinguish the homogeneous and heterogeneous pixels in textured areas effectively. Then, to enhance the filtering effect, it is necessary to take the adaptive threshold selection of similarity test (Simi-Test) into consideration. The simulated, low spatial resolution SAR580-Convair and high spatial resolution ESAR PolSAR image datasets are selected for experiments. We make a detailed quantitative and qualitative analysis for the filtered results. The experimental results have demonstrated that the proposed ANLM filter has better performance in speckle suppression and detail preservation than that of the traditional local and nonlocal filters.

## 1. Introduction

Polarimetric synthetic aperture radar (PolSAR) can measure the polarimetric characteristic of terrain echo and play an important role in remote sensing field [1]. PolSAR images acquired by airborne and spaceborne sensors can observe abundant terrain features used for many applications, for example, terrain classification [2,3,4], target detection [5,6], disaster monitoring [7,8], topography [9,10] and biomass estimation [11,12], etc. However, the application performance of PolSAR images is easily affected by speckle noise in coherent radar echo imaging systems because speckle noise seriously degrades the image quality [13,14]. Therefore, speckle filtering methods of PolSAR images have attracted the attention of many scholars and prompted many investigations in the past several decades.

With the purpose of maintaining a good balance between speckle removal and polarimetric characteristic preservation, many effective local or nonlocal speckle filtering methods have been proposed for PolSAR images [13,15,16,17]. The basic methodology of speckle filtering mainly consists of two key problems: (1) how to select homogeneous pixels, (2) how to estimate the central value from the selected pixels. Currently the second step has already been studied well, with many methods including average, weighted average [16,18], Lee filter [13,15,19], distributed Lee filter [20] and nonlocal reduced bias estimation (NLRB) [17] being proposed. To be specific, the weighted average is based on the similarity value and obtains the weighted maximum-likelihood result [16,18]; the Lee filter uses the intensity-based local linear minimum mean square (LLMMSE) estimate to prevent the strong multilook operation [13,15,19]; the distributed Lee filter combines the structure similarity and homogeneity similarity in a distributive way [20]; the NLRB estimate, a well-known and most reliable one, not only considers the similarity difference between homogeneous pixels but also gets an optimal basis-reduction result [17]. However, for the first problem of how to select homogeneous pixels there still exist many problems.

In local PolSAR speckle filtering, the way to select homogeneous pixels mainly involve the Oriented Windows (OW) method [13,21], Region Growing (RG) method [15,22], Point-Wise (PW) method [19,23,24], etc. It is noticed that the local methods mentioned above are basically based on pixel-wise similarity measures with intensities. Compared with the local speckle filtering methods, the nonlocal methods extend the point-wise similarity measure to patch-wise, which assumes that similar patches have similar center pixels. Obviously, the structure similarity measure can offer more accurate and robust performance in the selection of homogeneous pixels than point similarity measures, even when applied in a large search area (e.g., 15 × 15 windows). The nonlocal idea is firstly proposed by Buades et al., and since then most state-of–art speckle filtering methods draw on the structure similarity concept [25]. Deledalle et al. put forward an iterative weighted maximum likelihood denoising with probalistic patch-based weights [26] and then extended it to PolSAR [27] and interferometric SAR (InSAR) [28] images. Based on the complex Wishart distribution of PolSAR data, Chen et al. firstly proposed the likelihood ratio test as the adjustment criterion of similarity patches [16]. D’Hondt [24] and Torres [29] proposed a bilateral filtering method of PolSAR data based on stochastic and geodesic distance. Deledalle et al. offered a unified nonlocal framework for resolution-preserving (Pol)(In)SAR denoising, which provided the effect of efficient speckle reduction and good resolution preservation for subsequent applications [17]. Wang et al. proposed a finite mixture model (FMM) to adaptively fit the in-scene variation and then introduced a mixture-based NLM polarimetric filtering to better estimate the target statistics [30].

**Figure 1:**
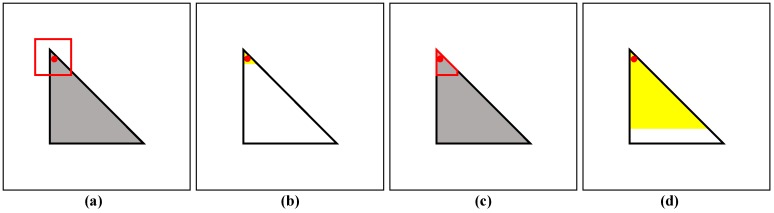
Homogeneous pixel selection of regular and SA matching patches near corner point, respectively. If it is used by the regular patches marked by red box in (**a**), the selected homogeneous pixels highlighted with yellow are very small in (**b**). On the contrary, based on SA matching patches in (**c**), the selection capability is flexible and the selected homogeneous pixels are much large in (**d**).

Previous nonlocal polarimetric filtering methods are mainly based on matching square patches to select the homogeneous pixels. However, the square patches have limited performance in textured areas (e.g., edges, corner points and stripes), because they contain such complex textured information. In this case, homogeneous pixels cannot be found or heterogeneous pixels are selected incorrectly in the search windows. In optical image denoising concepts, some methods have demonstrated the effectiveness of shape-adaptive (SA) patch matching, including BM3D with shape-adaptive principal component analysis (SAPCA-BM3D) [31] and non-local methods with shape-adaptive patches (NLM-SAP) [32]. However, it is difficult to obtain an accurate SA patch for a PolSAR image due to speckle noise. Currently, to solve this problem, the patch size must be small enough (e.g., 3 × 3 windows) to capture the textured information and prevent blurring, whose denoising performance is unfortunately still limited in textured areas. For example, in the ideal case, the capability of homogeneous pixel selection near corner point based on SA patches matching is much stronger than that based on square patches matching obviously in Figure 1. In result, the regular patches cannot find homogeneous pixels in textured areas; on the contrast, the result based on SA matching patches has many homogeneous pixels due to the adaptive capability. The second point is filtering parameters adaptation, and many efforts are focused on the optimum search windows size, matching patches size and pre-estimation scale [17,33,34]. Currently, the threshold selection of similarity test (Simi-Test) on patches matching has not attracted too much attention, and it is just set to a fixed value empirically, which ensures the suitable tradeoff between speckle reduction and detail preservation. However, in a homogeneous area, a larger threshold is preferred to achieve the maximum speckle reduction effect; in textured areas, a smaller threshold is beneficial for distinguishing homogeneous and heterogeneous pixels. For solving the above two problems, we propose the ANLM filter to improve the performance on homogeneous pixel selection more accurately and robustly. The proposed method based on the SA patches can match more homogeneous pixels successfully, especially in textured areas. Also, based on the estimated equivalent number of looks (ENL) with the NLRB method, an optimal threshold of Simi-Test can be found adaptively to perform a better denoising effect for every pixel in both homogeneous and textured areas. 

The paper is organized as follows: Section 2 illustrates PolLRT-CMRG method for constructing SA matching patches. Section 3 describes the main detailed steps of the adaptive polarimetric SAR filtering, including pre-estimation, nonlocal mean (NLM) estimate with SA patches matching, NLRB estimate from the selected homogeneous pixels and optimal selection of multiple estimations with multiple parameters. In Section 4, we simulate a 3-look PolSAR image dataset and compare the proposed ANLM filter to other traditional filters by the error analysis and detail preservation. Two low spatial resolution SAR580-Convair and high spatial resolution ESAR PolSAR image datasets are selected for the real experiments and we make a detailed analysis for the filtered results on speckle reduction and information preservation in Section 5. Finally, we draw the conclusions in Section 6.

## 2. The Construction of SA Matching Patches Based on the Proposed PolLRT-CMRG Method

In this section, we review the PolSAR image statistics. Then the proposed PolLRT-CMRG method combines the polarimetric likelihood ratio test for coherency matrices (PolLRT-CM) and the region growing (RG) method. It can effectively construct an adaptive-neighborhood (AN) for every pixel as SA matching patches.

### 2.1. PolSAR Images Statistic

For PolSAR images, the scattering matrix S usually can be used for describing the full polarmetric information of a single target. In case of linear horizontal and vertical polarization base, S is expressed as follows [1]:

(1) $$\;S=\left[\begin{array}{cc} S_{HH} & S_{HV}\\ S_{VH} & S_{VV} \end{array}\right]$$

With the reciprocal condition, $S_{HV}=S_{VH}$, S is also represented by a complex Pauli basis vector $k_{pauli}={\left[\begin{array}{ccc} S_{HH+VV} & S_{HH-VV} & 2S_{HV} \end{array}\right]}^{T}/\sqrt{2}$, where T denotes the matrix transpose [1]. And the total power SPAN of $k_{pauli}$ is obtained as:

(2) $$\;SPAN=\left(S_{HH+VV}^{2}+S_{HH-VV}^{2}+4S_{HV}^{2}\right)/2$$

For the distributed target, the polarimetric information should be expressed by the coherency matrix as follows [1,35]:

(3) $$\;T=\frac{1}{L}\overset{L}{\underset{i=1}{\sum }}k_{pauli,i}k_{pauli,i}^{*T}=k_{pauli,i}k_{pauli,i}^{*T}$$

where * donates the conjugate, L is the number of looks, and 〈·〉 donates the spatial or temporal operator.

Referring to Equation (3), let A=LT. The matrix A obeys the complex Wishart distribution, A ϵ W(q,L,Σ) with Σ=E(T), and the probability density function can be expressed as [1,35]:

(4) $$\;P_{T}^{\left(q,L,\Sigma \right)}\left(A\right)=\frac{{\left|A\right|}^{L-q}exp\left[-Tr\left(\Sigma^{-1}A\right)\right]}{K\left(L,q\right){\left|\Sigma \right|}^{L}}$$

where q is the dimension of the matrix A, Tr(·) denotes the trace of the matrix, and:

(5) $$\;K\left(L,q\right)=\pi^{\frac{q\left(q-1\right)}{2}}\overset{q}{\underset{j=1}{\sum }}\Gamma \left(L-j+1\right)$$

where Γ(·) is the gamma function.

We suppose that the matrices X and Y are independent and follow a complex Wishart distribution, respectively, i.e., X ϵ W(q,n,Σ) and Y ϵ W(q,m,Σ). Then their summation result also follows a complex Wishart distribution, i.e., X+Y ϵ W(q,n+m,Σ).

### 2.2. The Proposed PolLRT-CMRG Method for Constructing SA Matching Patches

In traditional nonlocal polarimetric filtering methods, square patches are used for matching and finding homogeneous pixels, but this has unsatisfactory performance in textured areas and hence has a very low filtering effect. These two SAPCA-BM3D and NLM-SAP filters in image denoising concepts have successfully obtained SA matching patches and good performance on speckle reduction. However, the PolSAR image is affected by strong speckle noise easily so that the above methods are difficult to apply to PolSAR images. Currently, in PolSAR filtering concepts, it has been demonstrated that the OW and RG methods can obtain local AN effectively, which have the potential of obtaining the accurate SA patches [13,15,21,22]. Especially, the intensity-driven adaptive neighborhood (IDAN) method based on RG method can obtain more flexible shape, and hence its result can preserve more tiny and flexible structures than that of the OW. However, these methods only using SAR intensity information seem to be unsatisfactory and unstable in complex scenes or affected by strong noise. Therefore, we combine the PolLRT-CM and RG methods to build up an accurate SA matching patches more robustly.

The test for equality of two complex Wishart matrices is firstly proposed in [36], which can be called PolLRT-CM. Supposing X and Y independently follow a complex Wishart distribution, i.e., $X\in W\left(q,n,\Sigma_{X}\right)$ with ${\hat{\Sigma }}_{X}=\frac{1}{n}X$ and $Y\in W\left(q,m,\Sigma_{Y}\right)$ with ${\hat{\Sigma }}_{Y}=\frac{1}{m}Y$. For application, the number of looks are usually equal for *X* and *Y*, i.e., n=m. The null hypothesis is $H_{0}:\Sigma_{X}=\Sigma_{Y}$, and the alternative is $H_{1}:\Sigma_{X}\neq \Sigma_{Y}$.

According to the probability density function of the matrices X, Y and X+Y, the polarimetric likelihood ratio test statistic can be expressed as:

(6) $$\;Q=\frac{2^{2nq}{\left|X\right|}^{n}{\left|Y\right|}^{n}}{{\left|X+Y\right|}^{2n}}$$

Meanwhile, we also take the logarithm as follows:

(7)  lnQ=n(2qln2+ln|X|+ln|Y|−2ln|X+Y|)

The approximate distribution function of lnQ can be computed as:

(8) $$\;P\left(-2\rho lnQ\leq z\right)≅P\left(\chi^{2}\left(q^{2}\right)\leq z\right)+\omega_{2}\left[P\left(\chi^{2}\left(q^{2}+4\right)\leq z\right)-P\left(\chi^{2}\left(q^{2}\right)\leq z\right)\right]$$

where:

(9) $$\;\rho =1-\frac{2q^{2}-1}{4nq}$$

(10) $$\;\omega_{2}=-\frac{q^{2}}{4}{\left(1-\frac{1}{\rho }\right)}^{2}+\frac{7}{96}\frac{q^{2}\left(q^{2}-1\right)}{n^{2}\rho^{2}}$$

It is noticed that the complex Wishart matrices are more similar when lnQ is closer to 0.

The PolLRT-CM method can be thought of as the growing principle of RG in the proposed PolLRT-CMRG method, which is more accurate and robust than the sigma test based on gamma distribution [15,19,22]. In the proposed PolLRT-CMRG method, it needs to set the maximum size of patches (MSP), the number of homogeneous pixels (NHP) in SA patches*.* The direct neighbors $x^{\prime }$ of the seed x can be the similar, if it meets the following aggregation test:

(11) $$\;-2\rho lnQ_{x,x^{\prime }}\leq Th_{\alpha },\;\;\;P\left(-2\rho lnQ\leq Th_{\alpha }\right)<1-\alpha \;\to Homogeneous\;Pixels$$

**Figure 2:**
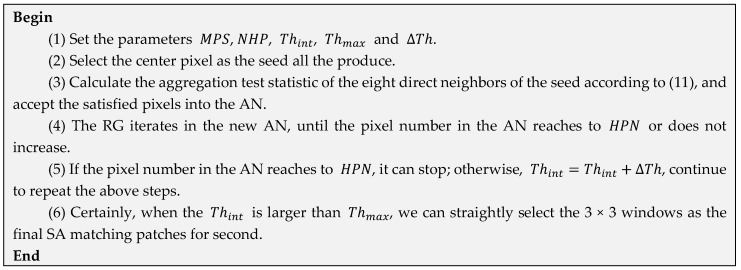
Flowchart of the proposed PolLRT-CMRG method for constructing SA matching patches.

Based on the framework of the traditional RG method, we propose an iterative method to refine the patches shape in Figure 2. It needs to set the initial threshold $Th_{int}$, the maximum tolerable threshold $Th_{max}$ and the increased interval value ΔTh. Inherited from the framework of the traditional RG method, the proposed PolLRT-CMRG method is shown in Figure 2.

In summary, the proposed PolLRT-CMRG method is an iterative process of gradual refinement. For every pixel in PolSAR images, the proposed method is performed to obtain rather accurate SA matching patches. Compared to the traditional IDAN filter, it takes the polarimetric information into consideration and selects PolLRT-CM method as the growing principle of RG, which is beneficial for the following step of homogeneous pixel selection, especially for the complex textured areas.

## 3. The Proposed ANLM Filtering for PolSAR Images

**Figure 3:**
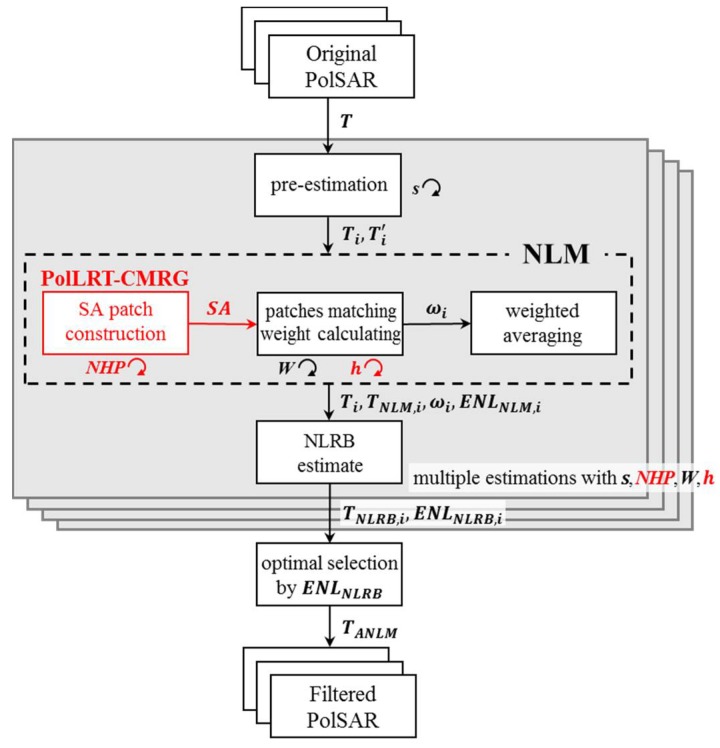
The flowchart of the proposed ANLM filter.

In our study, the proposed ANLM filter improves the unified framework for PolSAR images denoising and has better speckle reduction and information preservation performance (e.g., textured information and polarimetric characteristics) than the traditional nonlocal filters both in homogeneous and textured areas. The SA matching patches acquired by the proposed PolLRT-CMRG method mentioned in Section 2 can improve the ability of homogeneous pixel selection in textured areas more than the traditional nonlocal mean filters with square patches. The optimal threshold selection of Simi-Test can help to enhance the denoising effect in homogeneous areas and detail preservation in textured areas, respectively. As shown in Figure 3 the proposed ANLM filter contains four main parts. The details of each steps are explained in the following sections.

### 3.1. Pre-Estimation of Polarimetric Coherency Matrices with Reconstruction and Prefiltering

The nonlocal estimation methods usually involve polarimetric coherency matrix operations, e.g., determination operation in the known polarimetric likelihood ratio test for similarity of two matching patches (PolLRT-MP) [16,17,20]. However, it is worth noticing that when the number of look L is smaller than the matrix dimension q, the polarimetric coherency matrix T is singular, i.e., |T|=0 and it no longer follows the complex Wishart distribution. There exist some reconstruction methods enforcing full rank, and one of the best ways is to rescale the off-diagonal elements due to the ability to preserve full polarimetric information. The resulting coherency matrix $T^{\prime }$ of the proposed rescaling method can be obtained as follows:

(12) $$\;\{\begin{array}{c} \forall i=j,\;T_{i,j}^{'}=T_{i,j}\\ \forall i\neq j,\;T_{i,j}^{'}=\gamma T_{i,j},\;\gamma =\sqrt[{3}]{\min\left(L/q,1\right)} \end{array}$$

Due to the existence of strong speckle noise, low-contrast structures and characteristics are difficult to distinguish from surrounding areas. It is necessary to introduce some prefiltering method to reduce the estimation variance and improve the performance of similarity evaluation. In order to keep a good tradeoff between variance reduction and information preservation, the more robust and simple approach is needed to average some spatial samples, for example, it is performed by the Gaussian convolution in [17].

### 3.2. The NLM Estimate with SA Patches Matching

There exist lots of different statistical tests for similarity pixel selection, including polarimetric likelihood ratio tests, joint-likelihood criteria and geodesic distances, etc. One of the well-known similarity pixels selection method is the PolLRT-MP one described in [16,17,20]. The proposed PolLRT-MP method supposes that the coherency matrices of two matching patches satisfy the complex Wishart distribution independently firstly. Then the joint distribution can be considered as the product of each coherency matrix distribution within the matching patches. Hence, by evaluating the equality of corresponding pixels, the PolLRT-MP statistic can be expressed as:

(13) $$\;H=\underset{i\in W}{\prod }Q_{i}$$

where *W* is pixel sets within the matching patches. In the proposed ANLM filter, with the purpose of selecting more similarity pixels in textured areas, we select SA matching patches based on PolLRT-CMRG method mentioned in Section 2 as the *W* set. Then we can take the logarithm of Equation (13) and get:

(14) $$\;lnH=\sum_{i\in SA}lnQ_{i}=\sum_{i\in SA}n\left(2qln2+ln\left|X_{i}\right|+ln\left|Y_{i}\right|-2ln\left|X_{i}+Y_{i}\right|\right)$$

Likewise the two matching patches can be considered to be more similar when lnH is closer to 0. To obtain the PolLRT-MP threshold, we need to obtain the distribution function of lnH. In practice, we can select a homogeneous area, estimate the equivalent number of looks (ENL) and make statistics of lnH from the homogeneous area or simulated data based on the ENL.

The Simi-Test threshold and weight mapping are very important for nonlocal mean estimation. Generally, for the pixel x and $x^{\prime }$, the threshold parameter $h_{\alpha }\in {\mathbb{R}}_{+}$ of the PolLRT-MP method, is chosen to control the probability for the null hypothesis which supposes the two patches is similar [16,37]:

(15) $$\;lnH_{x,x^{\prime }}\geq \;h_{\alpha },\;\;\;P\left(lnH\geq h_{\alpha }\right)=\alpha \to Similar\;Patches$$

Then the mapping of weight from similarity $\omega \left(x,x^{\prime }\right)$ can be done with the kernel $exp\left(-lnH/h_{\alpha }\right)$. It is worth noting that it enforces the weight to 1 if and only if the coherency matrices of the selected pixel and center pixel are quite equal, i.e., lnH=0. But it is unreasonable because for PolSAR filtering the sum of two identical coherency matrices has no denoising effect acctually. Therefore, referring to [17], we suggest the more effective and simple weight mapping kernel as follows:

(16) $$\;\omega \left(x,x^{\prime }\right)=\{\begin{array}{cc} 1 & if\;x=x^{\prime }\\ exp\left(-\frac{\left|lnH_{x,x^{\prime }}-c\right|}{h}\right) & if\;x\neq x^{\prime } \end{array}$$

where $c=h_{\alpha }/2$, $h=h_{\alpha }/\left(2*k\right)$, k is the adjustment factor. The mapping definition ensures that the weight located in both threshold boundary is low and that in the middle is high, which is more consistent with the real condition.

After calculating the similarity $lnH_{x,x^{\prime }}$ and weight $\omega \left(x,x^{\prime }\right)$ over all pixels $x^{\prime }$ in a search windows W around the central pixel x, the NLM estimate performs a weighted averaging to a weighted maximum likelihood estimation:

(17) $$\;T_{NLM}\left(x\right)=\frac{\sum_{x^{\prime }}\omega \left(x,x^{\prime }\right)T\left(x^{\prime }\right)}{\sum_{x^{\prime }}\omega \left(x,x^{\prime }\right)}$$

It is noticed that $T\left(x^{\prime }\right)$ is the original coherency matrix, not the pre-estimation result $T^{\prime }\left(x^{\prime }\right)$ in Section 3.1, which can help to keep the original resolution of PolSAR images.

### 3.3. The NLRB Estimator from the Selected Homogeneous Pixels

The strong multilook operation, e.g., weighted averaging induced by nonlocal mean method mentioned above, may affect the edge information and polarimetric characteristic of the strong point target [13,15]. In order to reduce these effects, the linear minimum mean square estimator (LLMMSE) is first proposed in [38]. Deledalle et al. [17] combined the nonlocal mean estimation and improved LLMMSE method to suggest a more effective NLRB estimator, which is the best tradeoff between the nonlocal estimation and the original noisy coherency matrix. Therefore, the NLRB estimation in the proposed ANLM filter can be expressed as:

(18) $$\;T_{NLRB}\left(x\right)=T_{NLM}\left(x\right)+b\left(T\left(x\right)-T_{NLM}\left(x\right)\right)$$

where b is the weight factor. The b is close to 0, and the NLRB result means nonlocal mean estimation; otherwise, that is close to 1, and it means the original noisy coherency matrix.

Compared to the LLMMSE, the NLRB estimator achieves a better bias-variance tradeoff, which provides a lower bias and a higher variance. The following is the expression of the weight b:

(19) $$\;b=\underset{j}{\max}\left[\underset{}{\max}\left(0,\frac{var\left(I_{NLM}^{j}\left(x\right)\right)-I_{NLM}^{j}{\left(x\right)}^{2}/L}{var\left(I_{NLM}^{j}\left(x\right)\right)}\right)\right]$$

where j denotes a given polarimetric channel, $I_{NLM}^{j}\left(\cdot \right)$ is the intensity of j channel with NLM estimate, L is the ENL, $var\left(I_{NLM}^{j}\left(\cdot \right)\right)$ is the variance of $I_{NLM}^{j}\left(\cdot \right)$ which is calculated in [17].

### 3.4. Optimal Selection of Multiple Estimations with Multiple Parameters

In the nonlocal denoising procedure, we need to set several parameters artificially, mainly including the size of search window W, the scale of the pre-estimation s and the size of matching patches P. In homogeneous areas, large search windows (e.g., 15 × 15) are preferred for having better performance of speckle reduction. In textured areas, the size of matching patches needs to be small (e.g., 3 × 3) for preserving more details. The pre-estimation in enough large scale can improve the identification ability from homogeneous pixels. However, the Simi-Test threshold of PolLRT-MP is also an important parameter for PolSAR filtering, because it is well known that a larger threshold is preferred to reach the better effect of denoising in homogeneous area, and a smaller threshold is more appropriate to be set for identifying homogeneous pixels effectively in textured areas. Obviously, it is seen that the optimal threshold adaptation to local heterogeneous degree has the good potential of enhancing speckle reduction and detail preservation.

Deledalle et al. [17] have put forward the $ENL_{NLRB}$ calculation method based on NL-SAR estimate and it is a good indication of quality evaluation for PolSAR filtering. For the center pixel *x*, the $ENL_{NLRB}$ can be computed as follows:

(20) $$\;ENL_{NLRB}\left(x\right)=\frac{ENL_{NLM}\left(x\right)}{{\left(1-b\right)}^{2}+\left(b^{2}+\frac{2b\left(1-b\right)}{\sum_{x^{\prime }}\omega \left(x,x^{\prime }\right)}\right)ENL_{NLM}\left(x\right)}$$

where $ENL_{NLM}\left(\cdot \right)$ is the ENL calculated by the NLM method [17].

Referring to [17], we can set all parameters, including search window W, pre-estimation scale s, the number of homogeneous pixels NHP in SA patches and Simi-Test threshold h, calculate the estimation of NLRB and select the best estimation $T_{ANLM}$ according to the largest $ENL_{NLRB}$.

## 4. Experimental Results and Analysis of Simulated PolSAR Dataset

### 4.1. Data Sets Description and Experimental Settings

**Figure 4:**
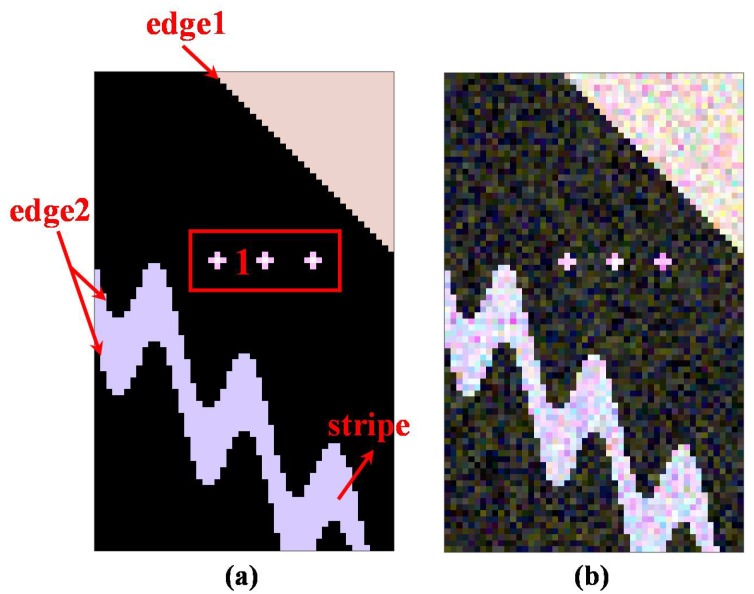
The Pauli basis composite RGB images. (**a**) Noise-free image. (**b**) Simulated image. The four marked areas are used for the subsequent quantitative evaluation of the denoising performance.

To demonstrate the effectiveness for the proposed ANLM method, we choose one simulated PolSAR dataset for quantitative evaluation. We use the Monte Carlo simulation method described in [1] for 80 × 50 3-look PolSAR images. The Pauli bias composite RGB images of the noise-free and simulated PolSAR data are also depicted in Figure 4. The simulated scene contains some typical structures, including strong point, linear edge, curved stripe and homogeneous areas.

For illustrating the performance of the proposed ANLM method on PolSAR image datasets, we perform comparisons with the refined Lee filter, IDAN filter, pretest filter and NL-SAR filter. According to the authors’ suggestions, the refined Lee filter have 7 × 7 edge-aligned neighborhood, the IDAN filter has an adaptive neighborhood of maximum size 50, pretest filter uses 15 × 15 search window and 3 × 3 matching patches. In the NL-SAR filter, the sets W of search window sizes, ℙ of matching patch sizes, and S are setting as the default, and the maximum size of search window $W_{max}$ is 15 × 15. The range of the multiple parameters in the proposed ANLM filter can be set as follows:

(1) W = {3 × 3, 7 × 7, 11 × 11, 15 × 15}
(2) S = {0, 1, 2}
(3) *MSP* = {5 × 5}, ℕℍℙ = {5, 9, 13, 17}, Th = {$Th_{0.01}$: ΔTh: $Th_{0.99}$}, ΔTh = ($Th_{0.99}-Th_{0.01}$)/10
(4) 𝕙 = {$h_{0.99}$: Δh: $h_{0.01}$}, Δh = ($h_{0.01}-h_{0.99}$)/4, *k* = 2

where ℕℍℙ and 𝕙 are the sets of homogeneous pixel number and Simi-Test threshold, respectively.

For comparing the algorithmic complexity of different nonlocal filters, we assume that the size of an PolSAR image is M×N. The pretest filter, one of a traditional NLM filter, has an computation complexity of order O(M·N·W·P), where *W* and *P* is the size of the search window and matching patch. The computation complexity of the NL-SAR filter is $O\left(M\cdot N\cdot W_{max}\cdot \left|\mathbb{P}\right|\cdot \left|S\right|\right)$ [17]. Since the number of pairs of the size of matching patches *P* and the scale of pre-estimation s are of the same order as the number of pixels in a patch, the complexity of the NL-SAR and pretest filters are at the same level. Similarly, the ANLM filter needs |W|·|ℕℍℙ|·|S|·|𝕙|  multiple estimations and selects the best estimation automatically, where ℕℍℙ and 𝕙 are the sets of the number of homogeneous pixels in SA matching patch and the Simi-Test threshold, respectively. The corresponding computation complexity is $O\left(M\cdot N\cdot W_{max}\cdot \left|\mathbb{N}ℍ\mathbb{P}\right|\cdot \left|S\right|\cdot \left|𝕙\right|\right)$. Therefore, considering the parameter adaptation of Simi-Test threshold, the algorithmic complexity of the proposed ANLM filter is higher than that of the NL-SAR and the pretest filters. To illustrate the effectiveness of the proposed ANLM more clearly, we also add one more experiment only using the SA matching patches, which sets the single Simi-Test threshold to be $h_{0.50}$ straightforwardly.

### 4.2. Quantitative Evaluation on Speckle Reduction and Detail Preservation

**Figure 5:**
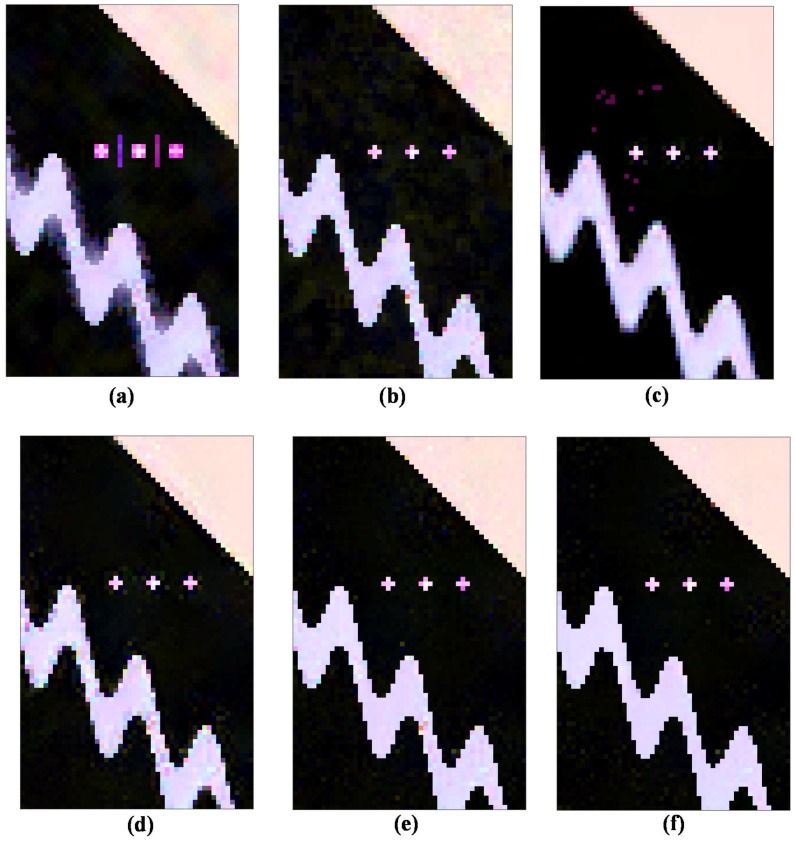
Denoising results of different methods for simulated PolSAR dataset. (**a**) Refined Lee filter. (**b**) IDAN filter. (**c**) Pretest filter. (**d**) NL-SAR filter. (**e**) The proposed ANLM with single threshold $h_{0.50}$. (**f**) The proposed ANLM with optimal threshold.

The simulated PolSAR images are processed by the aforementioned filters, and the corresponding filtering results are shown in Figure 5. Strong point targets and curved edges are blurred, and some false lines appear in the refined Lee filtered image. The IDAN filter is based on a pixel-wise approach to reconstruct the AN and preserves most basic structure details, but due to the limited denoising ability, there obviously exists residual speckle noise. The pretest nonlocal filter can suppress the speckle noise better, especially on the homogeneous areas, but due to the lack of a bias-reduction step, there exists some bias introduction around the strong point targets, and most edges are blurred strongly, especially curved ones. 

Compared to the pretest filter, the NL-SAR filter based on an NLRB estimate has better information preservation performance for edges and speckle reduction for homogeneous areas. However, based on matching with regular patches, the speckles are still visible on the straight and curved edges. From the filtered result in Figure 5e, the ANLM filter with single threshold significantly improves the denoising performance on the curved edge, and it is even much better than the IDAN filter. After considering the optimal threshold selection of Simi-Test, the proposed ANLM filter with optimal threshold can offer a more efficient reduction of noise in both the homogeneous and the textured areas. Also, the filtered details are clearer than that of NL-SAR method. Obviously, the proposed ANLM filter with optimal threshold shows the best speckle reduction performance.

To evaluate the error of the different filters quantitatively, we measure the filtering precision by the root mean square error (RMSE) between the real and filtered coherency matrices [39]:

(21) $$\;RMSET={\left(\frac{1}{MNq^{2}}\sum_{i}||T\left(x_{i}\right)-\hat{T}{\left(x_{i}\right)||}_{F}^{2}\right)}^{1/2}$$

where $x_{i}$ denotes the pixel index *i* in 2-D image, the matrix operator ${\left\|\cdot \right\|}_{F}$ denotes the Frobenius norm, $T\left(x_{i}\right)$ and $\hat{T}\left(x_{i}\right)$ are the estimated and real coherency matrix, respectively.

**Figure 6:**
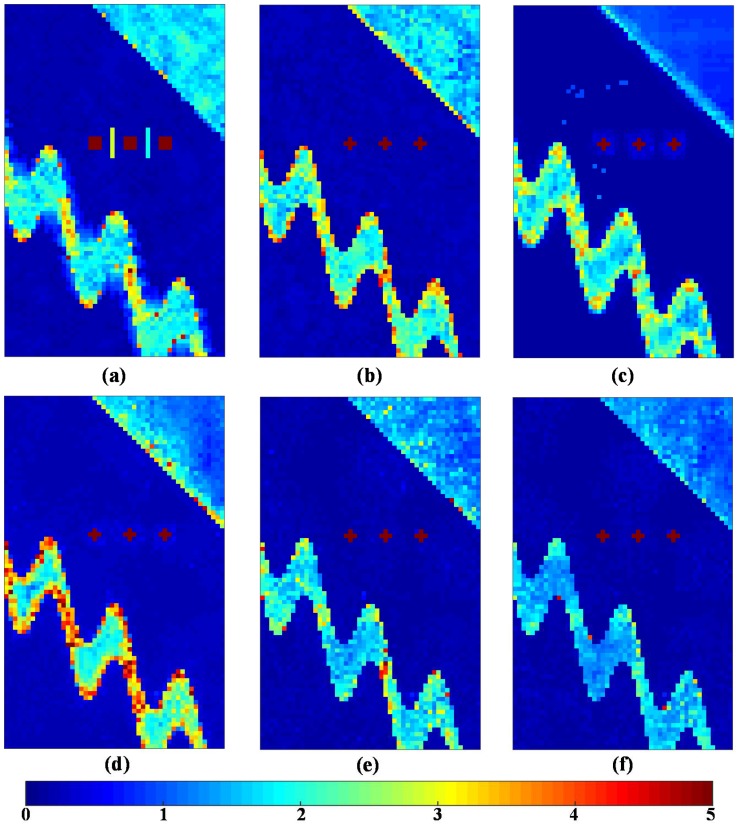
The RMSE of pixel-wise coherency matrices for different filtering methods. (**a**) Refined Lee filter. (**b**) IDAN filter. (**c**) Pretest filter. (**d**) NL-SAR filter. (**e**) The proposed ANLM with single threshold $h_{0.50}$. (**f**) The proposed ANLM with optimal threshold.

Figure 6 shows the point-wise filtered CM RMSE for different filtering methods. The pretest filter has the lowest error in homogeneous areas, especially in the upper right hand portion of the image. On the contrary, the points and edges are blurred and their surrounding errors are higher due to the strong multilook operation. From the results of the first four filters, there remain too many errors left on the curved edges, which shows the limitation of these filters on complex textured areas. The NL-SAR filter also has limited performance of speckle reduction in the stripe areas even though using the adaptive selection of the best parameters, including the scale of pre-estimate, the size of matching patch and search window. Compared with other filters, the filtered result of the ANLM with single threshold shows the SA matching patches has better potential of speckle reduction in complex textured areas. By means of the optimal threshold selection adaptively, the errors of the ANLM with optimal threshold are also reduced obviously in both the homogeneous and textured areas.

Then, for providing a quantitative analysis of the results further, Table 1 shows the standard deviations between the real and filtered CM on different filtering methods. We make many quantities indicators for different kinds of area shown in Figure 4, including RMSE, $RMSE_{\mathrm{stripe}}$, $RMSE_{\mathrm{edge}1}$, $RMSE_{\mathrm{edge}2}$ and $RMSE_{\mathrm{region}1}$ for evaluating the denoising performance of the whole scene, stripe, straight edge in the upper right, curved edge on two sides of stripe and region 1 around point targets, respectively. It is seen from the RMSE result that the ANLM method proposed in this paper leads to the best speckle reduction performance compared to that of other filters. The refined Lee filter leads to a low $RMSE_{\mathrm{edge}2}$ value of straight edge, but a high $RMSE_{\mathrm{region}1}$ around point targets. The IDAN filter has a low error value of $RMSE_{\mathrm{stripe}}$, $RMSE_{\mathrm{edge}1}$, $RMSE_{\mathrm{edge}2}$, and $RMSE_{\mathrm{region}1}$. Due to the over smoothing effect, the pretest filter has a low overall error value, but most details are blurred. Based on the square patches matching and NLRB estimate, the NL-SAR has the highest errors in the textured areas. The main reason is that it simply keeps the original pixel value for preserving the textured information and spatial resolution in the textured areas. The proposed ANLM filter has the lowest error value of all quality indications. It shows that the SA patches matching decreases the speckle noise in the textured areas effectively and the adaptive selection of the optimal Simi-Test threshold is also applied successfully.

## 5. Experimental Results and Analysis of Real PolSAR Dataset

Two classical datasets of real PolSAR images with low and high spatial resolution are used for further analyzing the effectiveness of the proposed ANLM filter as follows. It is also compared to the refined Lee filter, the IDAN filter, the pretest filter and NL-SAR filter. The corresponding parameters setting of these filters are the same as that of the previous simulation experiment.

### 5.1. Performance Evaluation Based on Low Spatial Resolution SAR580-Convair Data

**Figure 7:**
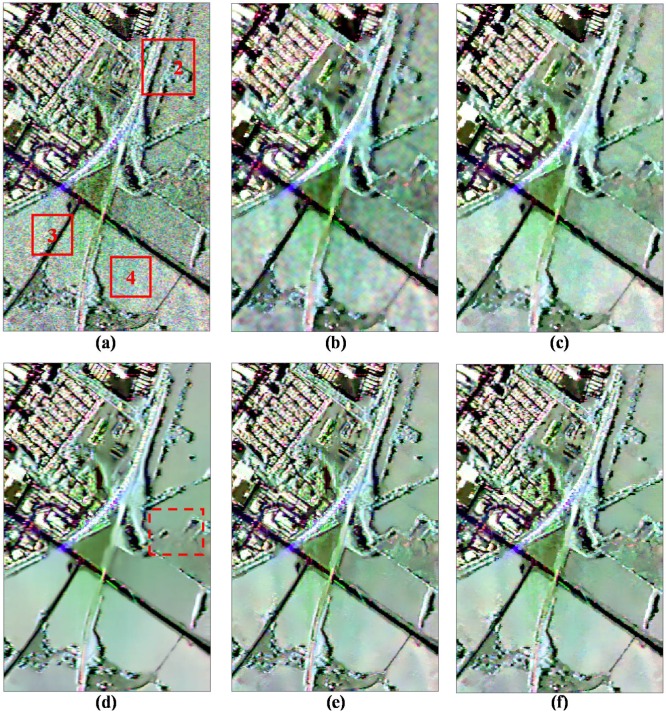
Denoising results of different filtering methods using SAR580-Convair C-band data. (**a**) Original data. (**b**) Refined Lee filter. (**c**) IDAN filter. (**d**) Pretest filter. (**e**) NL-SAR filter. (**f**) The proposed ANLM filter.

The first dataset is the SAR580-Convair C-band PolSAR multilook image whose resolution is 6.4 m in azimuth and 10 m in range, respectively. The original Pauli RGB composite image is shown in Figure 7a. The estimated ENL is 5.0 using the matrix trace moments (TM) [40], which is also based on the complex Wishart distribution.

Figure 7 shows the results of different filters for visual inspection. Even though the refined Lee filter (see Figure 7b) preserves most strong points and edges, these details have been blurred and the scallop effect appears in the homogeneous areas due to the edge-aligned windows. Figure 7c shows the result of the IDAN filter. We can see that many tiny details are smoothed and the speckle noise is visible in some areas for the IDAN filter. The pretest nonlocal filter shows the good speckle suppression ability in the homogeneous areas, but most textured structures have been smoothed or removed obviously, especially in the dashed boxes in Figure 7d. From Figure 7e,f, it can be seen that both the NL-SAR and the proposed ANLM filters exhibit similar and good performance in both speckle reduction and detail preservation. 

**Figure 8:**
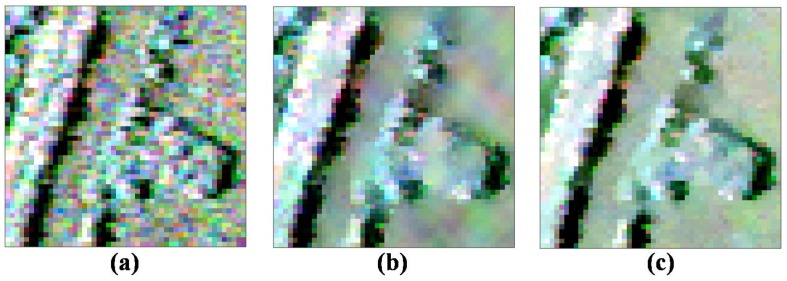

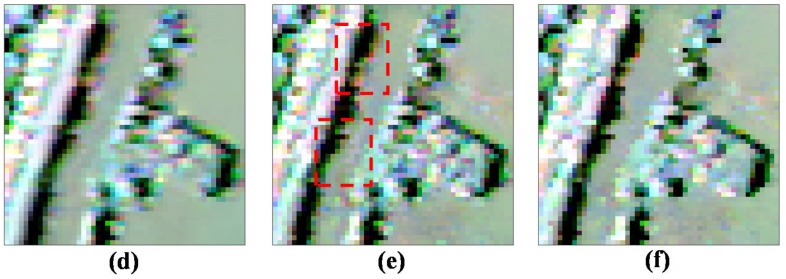
Enlarged details on the marked region 2. (**a**) Original data. (**b**) Refined Lee filter. (**c**) IDAN filter. (**d**) Pretest filter. (**e**) NL-SAR filter. (**f**) The proposed ANLM filter.

To analyze the denoising performance further, Figure 8 gives the enlarged images of the marked region 2 shown in Figure 7a. It is observed that the textured information of the NL-SAR and the proposed ANLM filters look much clearer than that of the other filters. However, there are still some speckle noises left on the edge and stripe marked by dashed box in Figure 8e after NL-SAR filter, and it just tends to keep the pixel value of original PolSAR image in textured areas. Compared with the NL-SAR filter, it is seen that the edge and stripe details are much clearer and speckle noise is not left visually with the proposed ANLM filter. Therefore, it has the potential of speckle reduction in textured areas.

It is necessary to evaluate whether the PolSAR filters affect the characteristics between polarimetric channels. The complex correlation just can measure the performance of preserving polarimetric information, and it can be expressed [14]:

(22) $$\;\rho_{i,j}=\frac{E\left(S_{i}S_{j}^{*}\right)}{\sqrt{E\left({\left|S_{i}\right|}^{2}\right)E\left({\left|S_{j}\right|}^{2}\right)}}$$

where i and j resprent two polarimetric channels.

We select the homogeneous region 4 marked in Figure 7a and estimate all possible complex correlations of the original and filtered images shown in Table 2, including $\rho_{hh+vv,hh-vv}$, $\rho_{hh+vv,hv}$ and $\rho_{hh-vv,hv}$. If the complex correlation of the filtered images is close to that of the original images, the corresponding filter has the good performance of information preservation. The pretest filter makes the original polarimetric characteristics far away from the original characteristics due to the strong multilook operation; on the contrary, other filters, including the refined Lee, IDAN, NL-SAR and ANLM filters, is based on the LLMMSE or NLRB estimate to keep the better tradeoff between the nonlocal estimate and the original images.

**Figure 9:**
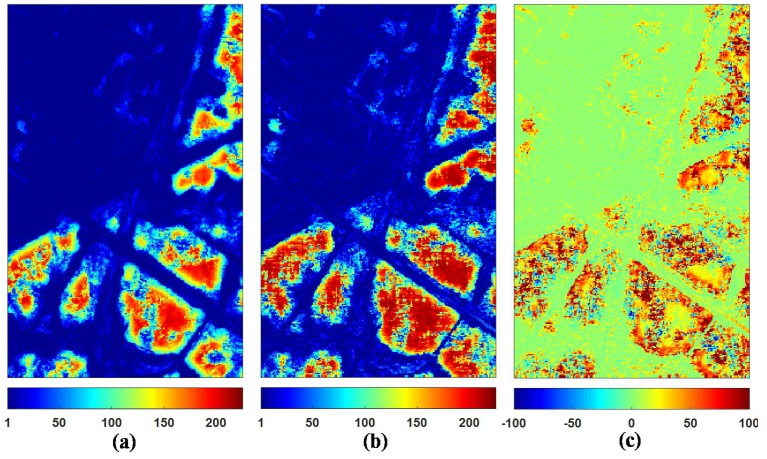
Comparison of the NL-SAR and the ANLM filters by the $ENL_{NLRB}$. (**a**) The NL-SAR filter. (**b**) The ANLM filter. (**c**) The $ENL_{NLRB}$ difference between the ANLM and the NL-SAR filters.

The following is the further analysis between the proposed ANLM filter and the NL-SAR filter. Both the two filters are based on the $ENL_{NLRB}$ of NLRB estimate to select the optimal parameter adaptively. The higher the $ENL_{NLRB}$, the better the speckle suppression. Therefore, we choose the quality indication $ENL_{NLRB}$ to evaluate the suppression effect of speckle noise for NL-SAR and ANLM filters. The $ENL_{NLRB}$ maps of NL-SAR and ANLM filters and its difference are shown in Figure 9. We can see that the proposed ANLM filter has higher $ENL_{NLRB}$ value than that of the NL-SAR filter on the whole. Table 3 presents the average $ENL_{NLRB}$ value obtained from the full scene and the marked regions 2–4 in Figure 7a to evaluate the denoising capability quantitatively. From the results of the regions 2 and 3, the filtering performance of the proposed ANLM filter in textured areas has been improved greatly. Also it is seen from the results of region 4 that it has the better speckle reduction effect on homogeneous areas.

### 5.2. Performance Evaluation Based on High Spatial Resolution ESAR Data

The second dataset is the DLR (German Aerospace Centre) ESAR L-band PolSAR data taken over the Oberpfaffenhofen test site in Germany in 1999, whose original resolution is 1.5 m in the range and azimuth directions, respectively. For equalizing the resolution of azimuth and ground range directions, we also make a two-look processing in azimuth. A region of 500 × 800 pixels is selected for the following experimental analysis. This PolSAR image has a very high spatial resolution and too many kinds of complex structures, including strong point targets, edges, lines, stripe and homogeneous areas. Therefore, it is difficult to keep a good balance between denoising and detail restoration for filters, which is suitable for filtering performance evaluation. The estimated ENL is 1.4 using the TM estimator.

**Figure 10:**
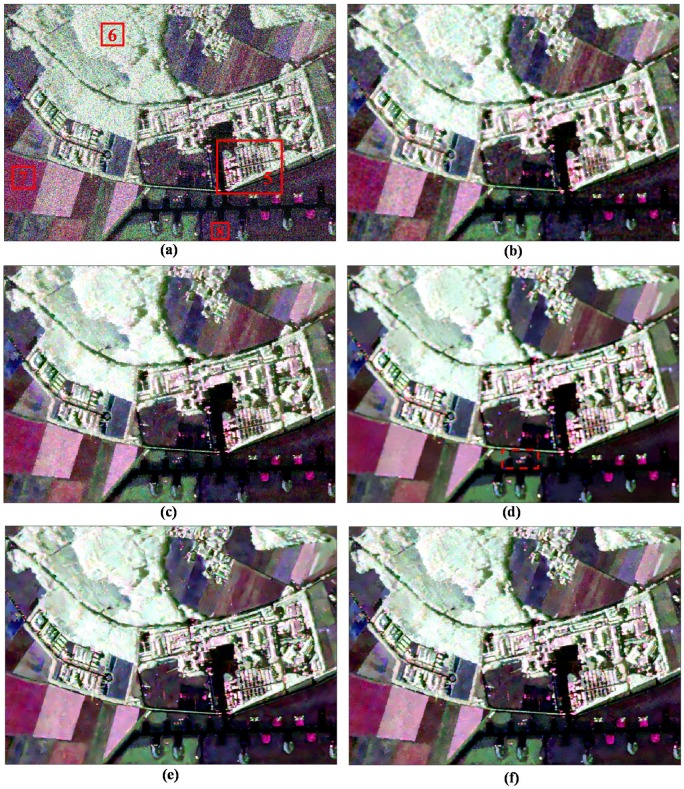
Denoising results of different filtering methods using ESAR L-band data. (**a**) Original data. (**b**) Refined Lee filter. (**c**) IDAN filter. (**d**) Pretest filter. (**e**) NL-SAR filter. (**f**) The proposed ANLM filter.

Figure 10 shows the visual assessment of the original data and filtered results by the above five filters. For the refined Lee filtered result shown in Figure 10b, the scallop effect in homogeneous area is obvious and some details are blurred. The IDAN filtered result (see Figure 10c) preserves most of the details, but the speckle noise in homogeneous area is visible. The pretest nonlocal filter (see Figure 10d) has a good performance in speckle noise reduction; however, the point targets are spreading obviously, especially in the dashed boxes in Figure 10d. From Figure 10e,f, it can be seen that both the NL-SAR and the proposed ANLM filters have similar and good performance in most areas.

**Figure 11:**
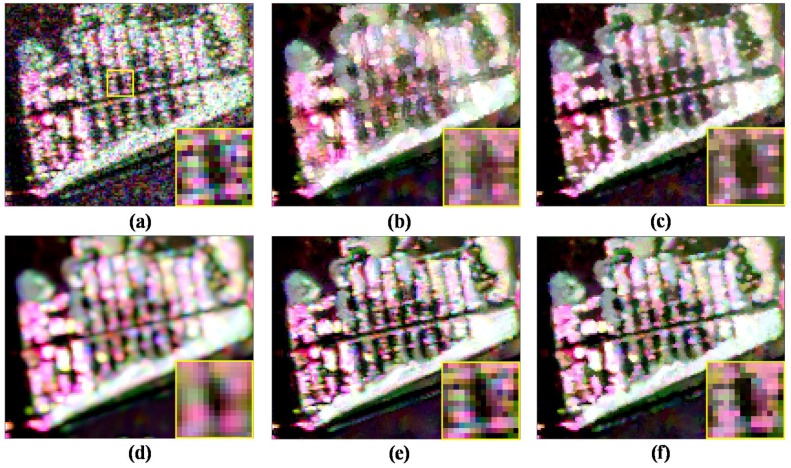
Enlarged details on marked region 5. (**a**) Original data. (**b**) Refined Lee filter. (**c**) IDAN filter. (**d**) Pretest filter. (**e**) NL-SAR filter. (**f**) The proposed ANLM filter.

Likewise, we select the enlarged image on the marked region 5 in Figure 10a to compare the denoising performance of different filters in Figure 11. Region 5 has too many kinds of surface features and the textured information is rather complex. It can be observed that the refined Lee filtered image has many false lines, the IDAN filtered image smoothes out many tiny details, and the pretest filtered image has lost most of the textured information. In the textured areas, the NL-SAR filter based on the square patches matching has low filtering effect, as it tends to keep the pixel value of original PolSAR data and has lost the filtering ability in fact for the textured areas. Especially, the yellow box image marked in Figure 11a of the NL-SAR filtered result and the original Pauli RGB image are very similar. 

This means that there is lots of speckle noise left after the NL-SAR filtering. Figure 11f shows the proposed ANLM filtered result. The edge in the enlarged image shown in Figure 11f is very clear and the noise around the image is well suppressed. Therefore, in textured areas, the ANLM filter can not only suppress the speckle noise greatly, but also preserve most of textured information well.

**Figure 12:**
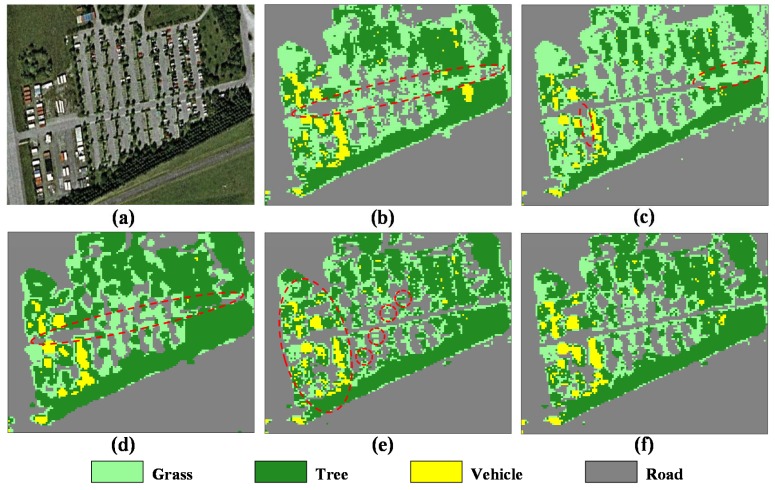
H/α−Wishart classification results of different filters. (**a**) Optical image. (**b**) Refined Lee filter. (**c**) IDAN filter. (**d**) Pretest filter. (**e**) NL-SAR filter. (**f**) The proposed ANLM filter.

One of the most important applications of PolSAR images is the terrain classification. Therefore, the classification result is also a good way to evaluate the preservation performance of textured and polarimetric information. In this paper, a widely used unsupervised H/α−Wishart classification method is selected for experiments [2]. Specifically, the number of pixels switching classes is 10%, the maximum number of iterations is 10, and the window size is 1 × 1 for verifying the validity of filters. Figure 10 shows H/α−Wishart classification results of different filters. The study area mainly contains grass, tree, vehicle and road. The optical Google Earth image acquired in 2001 is used for the reference image shown in Figure 12a. The refined Lee and pretest filters smooth too many details and line structures, for example, most of the road marked by the dashed ellipse in Figure 12b,d are obviously mistaken as grass or trees. The IDAN filtered results preserve more detail information, but some tiny details (e.g., roads) marked by the dashed ellipses in Figure 12c are lost. Though the NL-SAR filter preserves the most of details, its classification result presents the phenomenon of spatial discontinuity, especially for the vehicle and grass marked by the dashed ellipse in Figure 12e. Because the NL-SAR filter is based on the square patches matching and hence it is very difficult to find homogenous pixels from textured areas. Figure 12f shows the classification result by the proposed ANLM filter. The results show that the SA patch matching has good performance in distinguishing fours kinds of terrains, and the classification results preserve most of detail information and is distributed continuously in space.

**Figure 13:**
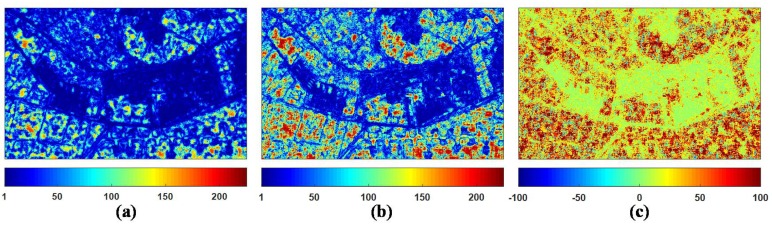
Comparison of NL-SAR and ANLM filters by $ENL_{NLRB}$. (**a**) NL-SAR filter. (**b**) ANLM filter. (**c**) The $ENL_{NLRB}$ difference between ANLM and NL-SAR filters.

**Figure 14:**
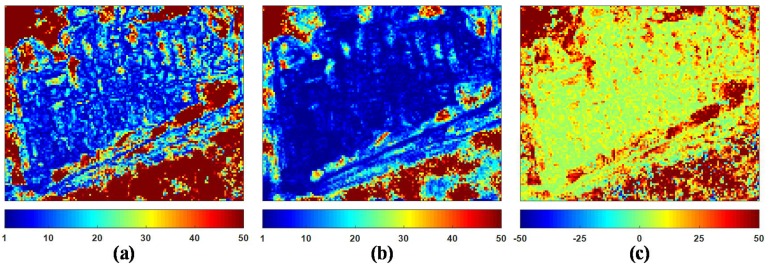
Enlarged $ENL_{NLRB}$ map on marked region 5. (**a**) NL-SAR filter. (**b**) ANLM filter. (**c**) The $ENL_{NLRB}$ difference between ANLM and NL-SAR filters.

Moreover, compared with the NL-SAR filter, it is necessary to highlight the advantage of the proposed ANLM filter by the $ENL_{NLRB}$. Figure 13 shows the $ENL_{NLRB}$ maps produced by the NL-SAR and the ANLM filters and their difference. On the whole, the $ENL_{NLRB}$ value of the proposed ANLM filter is much higher than that of the NL-SAR filter, and it means the proposed ANLM filter has much better performance in speckle suppression. Figure 14 also shows the enlarged $ENL_{NLRB}$ map of the NL-SAR and the ANLM filters and its difference on the marked region 5 in Figure 10a. Obviously, the proposed ANLM filter presents better capability of denoising in textured areas due to SA patches matching. Table 4 presents the average $ENL_{NLRB}$ value obtained from the full scene and the marked regions 5–8 in Figure 10a to evaluate the denoising capability quantitatively. From the result of the full scene, the proposed ANLM filter has improved the filtering performance of the unified nonlocal framework greatly introduced in [17]. In addition, it is observed from the results of the regions 6–8 that the speckle reduction effect on homogeneous areas of the proposed ANLM filter is also much better than that of the NL-SAR filter.

## 6. Conclusions

In this paper, an adaptive nonlocal mean filter (ANLM) for polarimetric SAR image data has been proposed based on shape-adaptive (SA) patches matching and optimal Simi-Test threshold selection. The proposed PolLRT-CMRG method combine the PolLRT-CM and RG methods, and obtains more homogeneous pixels in textured areas effectively for every pixel as SA patches. In addition, the Simi-Test threshold is an adaptive parameter to local structure based on the $ENL_{NLRB}$ value of the NLRB estimate, which can enhance the filtering effect both in homogeneous and textured areas. The simulated, the low spatial resolution SAR580-Convair and the high spatial resolution ESAR PolSAR datasets are selected for experiment. The experimental results have demonstrated the effectiveness of the ANLM filter on speckle reduction and detail preservation. The proposed filter can be applied to both low and high spatial resolution PolSAR images well. It is worth noticing that compared to other nonlocal filters for PolSAR images, the proposed ANLM filter has solved the problem of low filtering effect in textured areas while preserving the details.

## Figures and Tables

**Table 1:** The standard deviations between the filtered and real CM on different filtering methods.

Quality Indication	Simulated	Refined Lee	IDAN	Pretest	NL-SAR	ANLM with Single Threshold	ANLM with Optimal Threshold
RMSE	2.70	1.98	1.97	1.84	2.07	1.93	**1.79**
$\;RMSE_{\mathrm{stripe}}$	4.25	2.28	2.22	2.28	3.02	2.08	**1.62**
$\;RMSE_{\mathrm{edge}1}$	3.28	2.12	2.22	2.27	2.66	1.72	**1.38**
$\;RMSE_{\mathrm{edge}2}$	2.78	1.52	2.15	1.29	2.10	1.52	**1.23**
$\;RMSE_{\mathrm{region}1}$	0.43	1.76	0.20	0.23	0.30	0.17	**0.16**

**Table 2:** Quantitative comparison of different filters by all possible complex correlations on the marked region 4 in Figure 7a.

Complex Correlation	Original	Refined Lee	IDAN	Pretest	NL-SAR	ANLM
$\left|\rho_{hh+vv,hh-vv}\right|$	0.126	0.130	0.126	0.141	0.132	0.128
$arg\left(\rho_{hh+vv,hh-vv}\right)$	0.188	0.171	0.188	0.239	0.213	0.224
$\left|\rho_{hh+vv,hv}\right|$	0.036	0.033	0.036	0.034	0.033	0.034
$arg\left(\rho_{hh+vv,hv}\right)$	2.961	2.735	2.961	2.582	2.680	2.613
$\left|\rho_{hh-vv,hvv}\right|$	0.019	0.013	0.019	0.012	0.012	0.013
$arg\left(\rho_{hh-vv,hv}\right)$	−2.146	−2.271	−2.146	−2.434	−2.036	−2.220

**Table 3:** Quantitative comparison of the NL-SAR and the ANLM filters by average $ENL_{NLRB}$ on the full scene and the marked regions 2–4 in Figure 7a.

**Method**	**Full Scene**	**Region 2**	**Region 3**	**Region 4**
NL-SAR	35.0003	18.3173	52.7748	140.2214
ANLM	48.5872	31.6729	80.3525	172.6150

**Table 4:** Quantitative comparison of NL-SAR and ANLM filters by average $ENL_{NLRB}$ on full scene and marked regions 5–8 in Figure 10a.

Method	Full Scene	Region 5	Region 6	Region 7	Region 8
NL-SAR	35.7548	15.5849	37.0379	80.9794	90.0223
ANLM	67.0987	34.1219	74.5117	129.3978	150.0078
